# Looking at the smoking epidemic through the lens of population pyramids: sociodemographic patterns of smoking in Italy, 1983 to 2005

**DOI:** 10.1186/1478-7954-10-23

**Published:** 2012-11-28

**Authors:** Bruno Federico, Giovanni Capelli, Giuseppe Costa, Johan P Mackenbach, Anton E Kunst

**Affiliations:** 1 Department of Human Sciences, Society and Health, University of Cassino and Southern Lazio, Cassino, Italy; 2 Department of Public Health, Erasmus Medical Center, Rotterdam, Netherlands; 3 Department of Clinical and Biological Sciences, University of Turin, Turin, Italy; 4 Department of Public Health, Academic Medical Center, Amsterdam, Netherlands

**Keywords:** Smoking, Education, Time trends, Surveillance, Population pyramids

## Abstract

**Background:**

Surveillance systems often present data by means of summary measures, like age-standardised rates. In this study, we aimed at comparing information derived from commonly used measures of smoking with that presented in modified population pyramids (PPs), using the example of the diffusion of smoking in Italy over the past two decades.

**Methods:**

Data were derived from four National Health Interview Surveys carried out in 1983, 1990 to 1991, 1999 to 2000, and 2004 to 2005. After computing both age-specific and age-standardised rates of current, former, and never smoking, we constructed modified PPs by stratifying the male and female populations according to smoking status and educational level.

**Results:**

Modified PPs showed several features of the smoking epidemic in Italy that were not apparent from conventional surveillance techniques. First, they showed that the population of smokers is aging, with most current smokers in 2005 being males aged 25 to 39 and females aged 40 to 49, whereas in 1983 most smokers belonged to the youngest age groups. Second, they showed that in 2005 most smokers were found among subjects with middle and higher education, whereas two decades earlier most smokers were (male) subjects with the lowest education.

**Conclusions:**

Modified PPs were able to show how absolute numbers of smokers were distributed by age and sex, how these numbers varied between population subgroups, and how they changed over time. PPs may help provide information on past and future trends in the absolute number of smokers and in their sociodemographic characteristics, which may be missed using only traditional surveillance methods.

## Background

Tobacco smoking largely contributes to premature death and disease in developed countries [1]. It is estimated that tobacco caused about 5 million deaths in 2005, and the yearly death toll of smoking is expected to increase over the next 20 years [2]. This increase is a consequence of the diffusion that smoking had in the previous decades, because of the considerable time delay before smoking-related mortality rises [3].

In the UK, smoking reached its peak among men in the 1940s and among women two decades later [4]. A similar pattern was observed in the US and Northern Europe while southern European countries lagged behind in the progression of the epidemic [5-8].

Subjects at the top of the social hierarchy were the first to take up the habit of cigarette smoking, but afterwards the social pattern reversed, with higher smoking rates among the worst off [3]. Smoking is now associated with cultural, material, and social disadvantage in most Western countries [9]. Education, a widely used indicator of socioeconomic position, was negatively associated with smoking in several European countries. However, in Greece, Italy, Portugal, and Spain, the relationship between smoking and education was either weaker or inverse compared to northern European countries, with higher rates of smoking among the higher educated [10].

Monitoring the prevalence and distribution of smoking is essential in order to assess how smoking diffuses over time as well as to identify in which population groups smoking is more common. Monitoring is also needed to evaluate the effectiveness of tobacco control policies [11]. To these ends, surveillance systems are implemented at both the national and international levels. The Behavioral Risk Factors Surveillance System monitors smoking as well as other behavioural risk factors in the US [12], whereas examples of surveys that allow monitoring tobacco use in several countries are the Global Adult Tobacco Survey and the Global Youth Tobacco Survey [13].

Surveillance systems often present data by means of summary measure like age-standardised rates. Age-standardised prevalence rates are used in order to compare populations with different age structures [14]. It is also a common practice to display age-specific prevalence rates. However, neither the elaboration of standardised rates nor that of age-specific prevalence rates conveys information on the actual diffusion of the risk factor (that is, the absolute number of subjects exposed) and hence on the future burden of disease in the population. Health care systems and organizations require information from monitoring systems that helps them to allocate tobacco control resources where they are most needed [10]. In order to improve the reach of smoking cessation services, for instance, it is essential to know common sociodemographic characteristics of smokers. A simple analytical tool that can rapidly provide absolute numbers of smokers by sociodemographic characteristics may thus be of value.

Population pyramids (PPs), also known as age-sex pyramids, may be easily adapted to provide relevant information on such absolute numbers. A PP describes the age and gender structure of a population by means of two juxtaposed histograms, one for each gender. The absolute number of subjects in each age and gender subgroup is shown [15]. The shape of a PP is a function of both long-term trends in birth and death rates of a population, as well as of shorter-term events such as baby booms or wars [16]. By presenting the number of subjects according to age and gender, PPs also allow us to make predictions about the future age and gender structure of a population.

In this exploratory study, we compare information on the absolute number of smokers presented in modified PPs with that coming from commonly used smoking prevalence rates. We use the illustrative example of the diffusion of smoking in Italy, a country in which the epidemic has moved towards the later stages only in recent years [17,18]. Over the past decades, Italy witnessed a convergence in smoking rates between males and females, as well as a gradual shift from a positive association between smoking and socioeconomic position to a negative one, with the notable exception of older women. The focus of the present paper is the representation of trends in absolute numbers of smokers over the past two decades in the general population as well as by educational level.

## Methods

### Data sources

We used data deriving from four National Health Interview Surveys carried out in Italy in 1983 (n=89,000), 1990 to 1991 (n=67,000), 1999 to 2000 (n=140,000), and 2004 to 2005 (n=128,000). Each survey collected information on representative samples of the non-institutionalized Italian population using multistage sampling. Response rates were 90%, 89%, 87%, and 84%, respectively. Data on smoking were collected through the use of interviewer-administered questionnaires in the first two surveys and self-compiled questionnaires in the most recent ones.

The National Institute of Statistics (ISTAT) provided anonymous electronic datasets of the four surveys. From these datasets, we extracted data on age, gender, education, and smoking status for subjects aged 20 to 99. These individual-level variables were selected because they are strong determinants of smoking and are routinely collected in smoking surveillance systems [11]. On the basis of the highest educational level achieved by each subject, three educational categories were created: low (no education/primary education, International Standard Classification of Education [ISCED] levels 0–1), mid (lower secondary education, ISCED level 2) and high (upper-secondary/tertiary education, ISCED levels 3–8).

Individuals were classified as current smokers, former smokers, and never smokers on the basis of the question “Do you currently smoke?” Response options were “Yes”, “No and I never smoked,” and “No and I used to smoke.” Occasional smokers, as well as pipe and cigar smokers, were classified as never smokers. The same questions were used throughout the four surveys.

### Data analyses

Age (by 10-year categories) and gender-specific rates of current/former/never smoking were computed for subjects aged 20 to 99 at the time of each survey, taking into account individual survey weights provided by ISTAT. Combining these prevalence rates with population estimates, we were able to estimate the actual number of subjects in each sex and smoking category by single year of age. Population estimates of the age and gender distribution of the Italian population on January 1^st^ of each year were derived from the same institute [19,20]. Age standardisation was carried out using the direct method, with the Italian population in 2005 (males and females combined) as the reference population.

PPs were constructed by stratifying each bar of the two histograms that compose the PP (i.e., a single year of age per sex) according to individual smoking status (current, former, and never smoker). All statistical analyses were carried out using Stata 11. The code used to produce PPs was presented at the 2008 Italian Stata users meeting [21].

## Results

### Trends in smoking in the overall population

The age-standardised rates of current and ever smoking in Italy between 1983 and 2005 are shown in Table 1. Marked differences existed between men and women, with men showing higher rates of current and former smoking over the whole time period. Among men, there was a clear decline in the prevalence of current smoking between 1983 and 2005 (from 46.6% to 27.6%), whereas the prevalence of ever smoking (current and former smokers combined) declined to a much smaller extent (from 63.9% to 60.4%). Among females, current smoking prevalence was nearly stable over time, at a figure of about 17%, while the proportion of ever smokers increased from 20.5% to 31.9% over the same period.

**Table 1:** Age-standardised rates (%) of current, former, and never smoking among Italian males and females

	**Males**			**Females**		
	**Current smokers**	**Former smokers**	**Never smokers**	**Current smokers**	**Former smokers**	**Never smokers**
1983	46.6	17.3	36.1	17.8	2.7	79.5
1990	37.2	27.4	35.4	16.9	6.8	76.3
2000	31.5	30.9	37.6	17.8	12.6	69.6
2005	27.6	32.8	39.6	16.8	15.1	68.1

**Figure 1:**
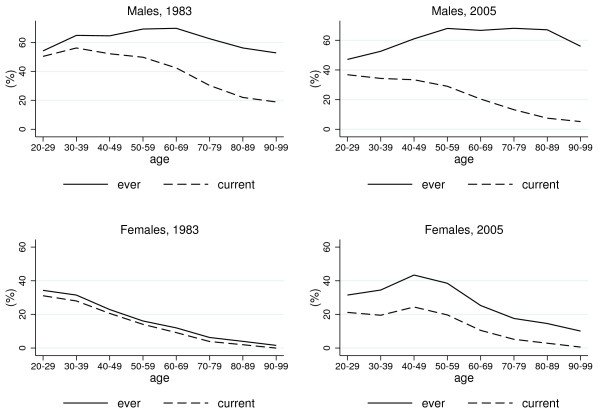
Age-specific rates of current and ever smoking in 1983 and 2005 among Italian males and females.

The age-specific rates of current and ever smoking in 1983 and 2005 are shown in Figure 1 separately by sex. Among males, the ever smoking rate, which reflects smoking uptake, did not markedly differ by age in 1983, whereas current smoking was more prevalent among young age groups than at older ages. On the other hand, among females there was a strong negative association between smoking and age in the same year, with both current and ever smoking rates clearly declining with age. The age profile of current and ever smoking were almost identical for females in 1983. Differences between current and ever smoking rates enlarged from 1983 to 2005 among both males and females. In 2005, current smoking rates among males decreased with age, whereas among females they peaked at ages 40 to 49.

**Figure 2:**
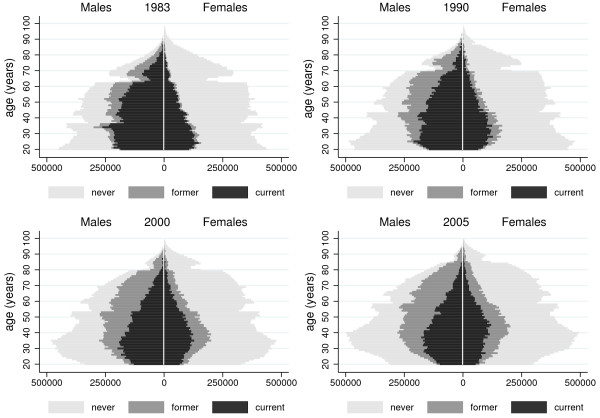
Population pyramids displaying the smoking status of Italian subjects in 1983, 1990, 2000 and 2005.

The modified PPs for year 1983, 1990, 2000, and 2005 are shown in Figure 2. Black, dark gray, and pale gray bars represent the absolute number of current, former, and never smokers, respectively, by single year of age. All pyramids have a very irregular shape, with abrupt ups and downs. A clear sex asymmetry in smoking habits was evident in 1983, with the vast majority of ever smokers being males, but this asymmetry tended to reduce over time. The graphs show that there was a decrease over time in the number of current smokers among males as well as a gradual increase in the number of former smokers among both males and females. The largest number of male current smokers was found among subjects aged 20 to 34 in 1983 and among subjects aged 25 to 39 in 2005. On the other hand, the largest number of female current smokers was found among subjects aged 20 to 29 in 1983 and among subjects aged 40 to 49 in 2005.

### Trends in smoking by educational level

**Figure 3:**
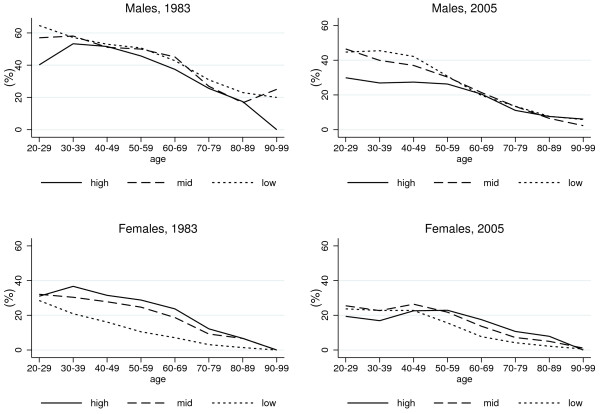
Age-specific rates of current smoking in 1983 and 2005 among Italian males and females with high, mid and low educational level.

**Figure 4:**
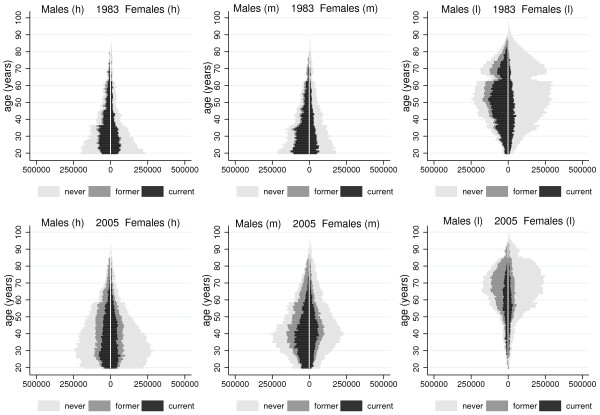
Population pyramids displaying the smoking status of Italian subjects with high (h), mid (m) and low (l) educational level in 1983 and 2005.

The relationship between education and smoking, and how it evolved over time, is shown in Figure 3 and Figure 4. In 1983, low-educated males had higher rates of current smoking than higher educated subjects, while the opposite was true among females (Figure 3). Twenty years later, educational differences in smoking increased among males in the younger age groups whereas among young females there was a reversal of the educational gradient, from positive to negative. Among women in the older age groups the positive association between smoking and education reduced.

The modified PPs in 1983 and 2005 are shown in Figure 4, separately by level of education. In both years, PPs have a large base and a narrow top for the highest educated while they take the shape of a spinning top (or a tornado) in the case of the lowest educated. In 1983, most current smokers were lowest educated males; a larger number of current smokers was found in the younger age groups among the higher educated (for both males and females), and in the age range 40–59 among the lowest educated (for males only). In 2005, the largest number of current smokers was found among subjects with lower secondary education, with a peak at age 30–39 among males and 35–44 among females. Among the highest educated a larger number of current smokers was found in the younger age groups, whereas comparatively few current smokers were in the lowest educational category. The PPs show that the number of low educated smokers rapidly decreased over time, while this was not the case for the number of higher educated smokers.

## Discussion

This study is mainly limited by the use of cross-sectional surveys. This implies that nonresponse and underreporting of smoking may affect our findings. However, nonresponse rates were generally small in the Italian National Health Interview Surveys, and self-reports of smoking are considered adequate [22]. Several studies described the evolving epidemics of smoking in Europe using similar surveys [6,10,17,18].

A good graph tells a story. Although the PP is a snapshot of basic demographic data, it tells the story of how the structure of an entire population is changing over time. Both long-term trends and sudden changes in fertility and mortality rates influence the shape of the PP. For instance, the marked reduction observed at the bottom of the PP between 1983 and 2005 was caused by declining fertility rates during the 1970s and 1980s in Italy [23], whereas the deep incisions were caused by catastrophic events, such as World Wars I and II. In the case of the lowest educated, the bottom of the PP decreased even more rapidly because of the upward shift in education observed in the second half of the 20^th^ century in Italy among young adults, following several reforms in the educational system [24].

To the best of our knowledge, hardly any use has been made of PPs outside the realm of demography. PPs were used to describe marital status according to age and sex in the UK [25], but no previously published study displayed health data using the PP. The graphical representation that we propose, which is both highly informative and easily understandable, may be of high relevance for policymakers. Graphs also provide a long-lasting memory to the reader compared to tabular representations [26].

PPs convey information that is not provided by common surveillance methods. First, the large asymmetry between males and females in smoking behaviour in 1983 and the fact that smokers were mostly young adults are both more visible with the PPs than with traditional representations. Absolute numbers are sometimes more relevant than relative numbers (i.e., rates) from a public health point of view. This applies to smoking cessation services, which need to plan their resources and activities according to the number of smokers. Population subgroups with the largest number of smokers do not necessarily have the highest prevalence rate of smoking. In the case of males in 2005, the age profile of the number of current smokers and that of current smoking rates did not exactly match: males who were 80 to 89 years old in 2005 had the highest rates of ever smoking, but the absolute number of these subjects was extremely small compared to other age groups.

Second, changes over time in the age and sex distribution of smokers become evident when PPs are made for different time periods: these changes are the result of both the underlying demographic processes (fertility and mortality) as well as the dynamic processes that regulate the flow of subjects into and out of the “pool” of smokers (i.e., initiation and cessation). With the use of PPs, we were able to show that, between 1983 and 2005, the population of smokers had a less skewed age distribution.

Third, PPs may be useful for comparing population subgroups that strongly differ in their age distribution. Stratifying the PPs by educational level, we were able to show that in 1983 most smokers were found among middle-aged males with the lowest education, whereas two decades later most smokers were found among young adults with higher educational levels. In the case of education, the story told by the PP is rather different compared with that displayed by the age-specific rates of current smoking: an inspection of these rates indeed suggests that, among young adults, the educational gap in smoking increased over time among young males, and that there was a reversal of the association between smoking and education among young females. Policy interventions aiming at reducing smoking behaviour within young adults with the lowest educational level, which is the subgroup with the highest smoking rate, would in fact provide only minor public health benefits, because there were very few smokers in this group, especially in 2005.

Finally, PPs may inform on future trends in the absolute number of smokers. PPs made clear that the population of smokers is likely to be aging within the next 20 years in Italy, as a result of the inevitable decline in the number of people younger than 40 years. Moreover, PPs made clear that, within about 20 years, smokers with a low educational level will start to be extinguished and they will be replaced by smokers with higher levels of education.

This study has a few implications for tobacco control policies. The first one relates to smoking surveillance, which is one of the pillars of tobacco control [11]. In addition to monitoring population groups with the highest prevalence of smoking, it may be useful to identify those that have the largest numbers of smokers. PPs help achieve this objective by combining, for each population group, information on trends over time of smoking patterns with information about trends in the fundamental demographic processes. The second implication relates to smoking cessation initiatives: in order to respond to the largest absolute health needs, cessation services in Italy should be directed at mid-educated male and female smokers in their 30s and 40s.

## Conclusions

Modified PPs are able to show how absolute numbers are distributed by gender and age, how these numbers change over time, and how they vary between population subgroups. We argue that the visual information provided by the modified PP make it a natural complement in surveillance to other statistical methods, such as the presentation of age-specific and age-standardised rates. We also suggest that this graphical representation may be of value to show trends over time in the absolute number of people exposed to other risk factors or health conditions, such as overweight and obesity, alcohol abuse, or physical disability.

## Competing interests

The authors declare that they have no competing interests.

## Authors’ contributions

BF and GCapelli conceived the study and they performed statistical analyses. GCosta provided data and helped in the interpretation of the study findings. AEK and JPM participated in the design of the study and they critically revised the manuscript. BF drafted the manuscript. All authors read and approved the final manuscript.
